# Pixel-Grouping G(E) Functions for Estimating Dose Rates from Unknown Source Distributions with a Position-Sensitive Detector

**DOI:** 10.3390/s23104591

**Published:** 2023-05-09

**Authors:** Hojik Kim, Junhyeok Kim, Jisung Hwang, Kilyoung Ko, Gyuseong Cho

**Affiliations:** Department of Nuclear and Quantun Engineering, Korea Advanced Institute of Science and Technology, 291, Daehak-ro, Yuseong-gu, Daejeon 34341, Republic of Korea; hojic90@kaist.ac.kr (H.K.); covent17@kaist.ac.kr (J.K.); jshwang93@kaist.ac.kr (J.H.); coltom@kaist.ac.kr (K.K.)

**Keywords:** G(E) function, spectrum-to-dose conversion coefficient, position sensitive detector (PSD), cadmium zinc telluride (CZT), ambient dose equivalent

## Abstract

Estimating accurate radiation doses when a radioactive source’s location is unknown can protect workers from radiation exposure. Unfortunately, depending on a detector’s shape and directional response variations, conventional G(E) function can be prone to inaccurate dose estimations. Therefore, this study estimated accurate radiation doses regardless of source distributions, using the multiple G(E) function groups (i.e., pixel-grouping G(E) functions) within a position-sensitive detector (PSD), which records the response position and energy inside the detector. Investigations revealed that, compared with the conventional G(E) function when source distributions are unknown, this study’s proposed pixel-grouping G(E) functions improved dose estimation accuracy by more than 1.5 times. Furthermore, although the conventional G(E) function produced substantially larger errors in certain directions or energy ranges, the proposed pixel-grouping G(E) functions estimate doses with more uniform errors at all directions and energies. Therefore, the proposed method estimates the dose with high accuracy and provides reliable results regardless of the location and energy of the source.

## 1. Introduction

Radiation is being widely used in various fields such as medical and nondestructive testing (NDT) and nuclear power plants, which has raised the importance of radiological protection for workers. Therefore, position-sensitive detectors (i.e., PSDs) are gaining attention as they can estimate the position of a radiation’s source from a reaction’s location and identify the nuclear species in the measured spectrum [1]. In addition to identifying a radiation’s source, estimating its dose in real time to protect workers from radiation exposure remains essential. Accordingly, the International Commission on Radiological Units and Measurements (ICRU) defined an ambient dose equivalent parameter {H*(10)} for measuring the radiation doses of high-penetration radiations such as gamma rays and neutrons [2,3]. H*(10) is defined as the dose equivalent in the expanded and aligned radiation field at a depth of 10 mm in the ICRU sphere. However, estimating the ambient dose equivalent H*(10), which is a measurable quantity, is difficult because it is impossible to make a detector exactly similar to the ICRU sphere. If the detector material is not tissue-equivalent, the response may differ depending on the energy of the radiation, resulting in incorrect dose estimation. Particularly, because many detectors (e.g., NaI(Tl), LaBr3(Ce), HPGe, and cadmium zinc telluride (CZT)) are denser than tissue, they overestimate the H*(10), especially at low energy [4,5,6,7].

Based on the above issues, two main methods were previously introduced for estimating dose: the unfolding method and the spectrum-to-dose conversion coefficient (i.e., the G€ function). The unfolding method involves extracting the gamma fluence from the measured spectrum and then calculating the dose by multiplying the fluence with the conversion coefficient [8]. However, the unfolding method has inherent noise that occurs during the unfolding process, and complexity of process [9]. The G(E) function can directly convert the measured spectrum into dose without any additional steps [10,11,12,13]. Nevertheless, the G(E) function has the essential limitation of significant errors at low energy. To overcome this limitation, a method was proposed to optimize coefficients using the ADAM method [5,13]; however, it is difficult to improve the difference in response depending on the direction of incidence of gamma rays. Nonspherical detectors cause the error of dose estimation depending on the direction of incidence of gamma rays, making them unreliable for dose estimation in most contamination situations with an unknown source distribution [14]. Hence, another attempt to improve dose estimation accuracies by employing two detectors was made, but this method had the drawback of a heavier system [15]. Furthermore, although combining the G(E) function with Gaussian processes increased reliability by providing the mean and standard deviation, this approach did not improve the accuracy of dose estimation [16].

Considering the above limitations, this study proposes pixel-grouping G(E) functions (i.e., G(E)_PG_ functions) to estimate doses, using multiple G(E) functions within the detector and position-sensitive properties. First, three rules were established to divide the inside of the detector into groups. To evaluate and compare with the conventional G(E) function (i.e., G(E)_C_ function), four pixel-grouping G(E) models (i.e., G(E)_PG_ models) and one conventional G(E) model (i.e., G(E)_C_ model) were determined. To optimize the coefficients of G(E)_PG_ models and evaluate the accuracy of dose estimation, an MCNP simulation dataset with randomly generated directions and energies was generated. Likewise, a dataset was generated to optimize the coefficients of the G(E)c model, and the means and standard deviations of the dose estimation was evaluated at an unknown source distribution. In addition, we verified the practical accuracy of G(E)_PG_ functions’ dose estimation through experimental data for six angles of three gamma sources (i.e., Cs_137_, Eu_152_, and Co_60_). Lastly, we investigated the role of each G(E)_PG_ function based on the gamma rays’ energies and incident directions.

## 2. Materials and Methods

### 2.1. Pixel-Grouping G(E) Function

The G(E) function is the spectrum-to-dose conversion coefficient used to correct the difference in attenuation coefficients depending on energy between tissue-equivalent materials and other materials (e.g., semiconductor, scintillator). Although previous studies have shown that the G(E) function estimates doses with relatively high accuracy, two limitations have, however, been observed. First, dose estimations using the G(E) function produce significant estimation errors at low energies. Second, the shape of nonspherical materials can affect responses, depending on the radiation’s incidence direction.

A detector is a device that identifies differences and obtains desired information through them. Most of the previous studies estimated the dose using the G(E) function with a device that only records energy (i.e., spectroscopy) with a single detector [10,11,12,13]. As the form of the G(E) function is not fixed, any function that best estimates the dose rate should be used. Power series of functions are suitable for mimicking various forms of functions, and, empirically, the power series of logE for G(E) function is the most widely used because it can estimate the dose rate with high accuracy [17].

(1) $$G\left(E\right)=\sum_{K=1}^{K_{MAX}}A(K){\left(\log E\right)}^{K-M-1}$$

Unfortunately, it is difficult to accurately estimate doses with such a device since the G(E) function cannot recognize direction-based differences. Nevertheless, with PSDs that can determine the location of the interaction and energy, doses can be estimated more accurately by dividing the detector area into multiple regions, using multiple G(E) functions for each region. In other words, the G(E) functions assigned to each divided region inside the detector can estimate dose by correcting each other based on the differences in the detector’s internal response caused by the two variables energy and direction.

**Figure 1:**
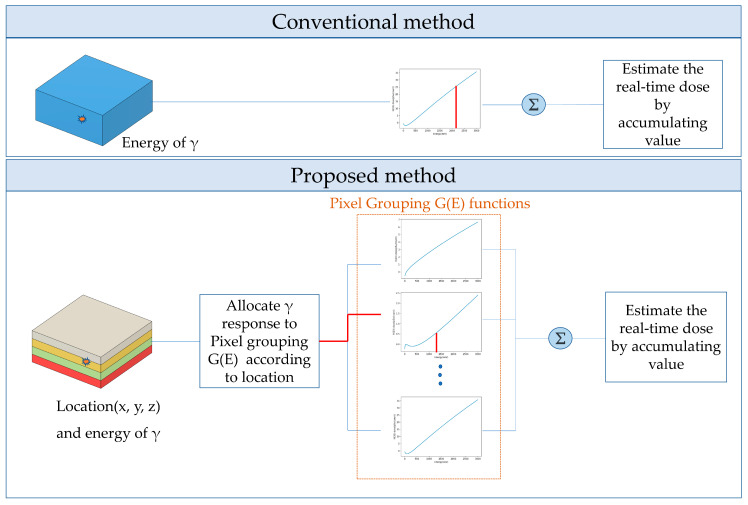
Schematic of the real-time dose estimation using pixel-grouping G(E) functions compared with the conventional G(E) function.

Figure 1 shows a schematic of the proposed methods. In the conventional method, the dose estimation involves accumulating the value of the G(E) function corresponding to the energy deposited in the detector. On the other hand, the proposed method, pixel-grouping G(E) functions (i.e., G(E)_PG_ functions), determines the group corresponding to the location of the interaction and estimates the dose by accumulating the value of the G(E)_PG_ function corresponding to the energy deposited in that group:

(2) $$D_{PG}=\sum_{p=1}^{P}\sum_{i=1}^{N}M_{p}(E_{i})\sum_{K=1}^{K_{MAX}}A_{p}(K){\left(\log E_{i}\right)}^{K-M-1}$$

where p is the number of divided groups, $M_{p}$ is the pulse height distribution per second measured from the group p, and $A_{p}$ is the coefficient of the G(E)_PG_ functions of the group p. When dividing the groups inside PSDs, rules to minimize unnecessary divisions should be established because the $K_{MAX}$ × p variable needs to be optimized.

- Rule 1: If the proportions of the visible surfaces remain unchanged depending on the incidence direction, they should be grouped into one.
- Rule 2: Grouping should be depth-based from the surface.
- Rule 3: A geometrically symmetric structure must be divided into equal shapes.

**Figure 2:**
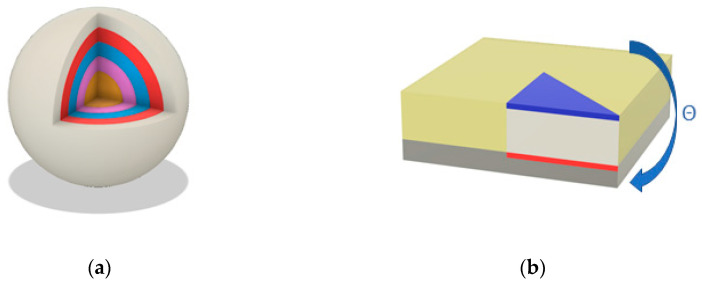
Example of a virtually divided position-sensitive detector (i.e., PSD) according to the rules; (**a**) a sphere shape of PSD; (**b**) a rectangular prism shape of PSD with the detector system.

For a sphere, as shown in Figure 2a, there is no difference in detector response depending on the direction; thus, the direction can be ignored. Therefore, we merged the surfaces into one group according to Rule 1. If the radiation energy is low, most of the reactions occur on the surface of the detector, and as the energy increases, the detector responds evenly throughout. Therefore, according to Rule 2, the G(E)function was thus grouped toward the depth direction from the surface, increasing the accuracy of dose estimation. Through Rules 1 and 2, Rule 3 had naturally been satisfied.

Figure 2b represents the simplified view of a rectangular prism detector and its detector system. According to Rule 1, as the direction of θ changed with the ratio of the visible surfaces for the top and side surfaces, they were separated. Additionally, as shown in Figure 2b, the radiation attenuates on the bottom surface because of the detector system, resulting in a change in area, so it needs to be divided as well. According to Rule 2, it needs to be divided in the depth direction from the surface. Finally, according to Rule 3, once the divided group of the triangular prism is determined, the remaining parts can be constructed symmetrically.

### 2.2. Experimental Setup and Data Generation

**Figure 3:**
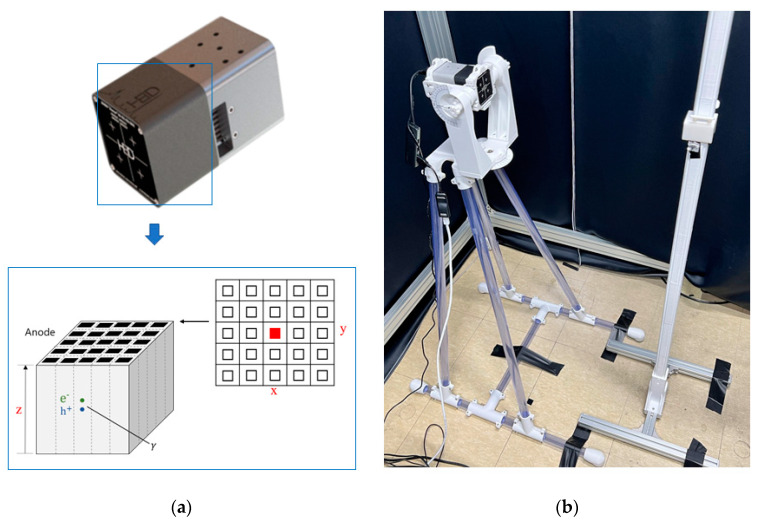
The cadmium zinc telluride (CZT) detector for experiment; (**a**) CZT detector module (H3D, M400) with the principle of PSD. (**b**) Experimental setup.

Unlike the HPGE detectors, as the CZT detector is semiconductor-based, it can operate at room temperature, and can maintain its performance with an air-cooling system. It has recently gained attention, especially due to its portability. In addition, the CZT detector has a high energy resolution and density of 5.8 g/cm^3^, making it extremely responsive and particularly useful for detecting high-energy gamma rays. The CZT detector (H3D, M400) detects 10 μCi Cs_137_ at 1 m (~3 μR/h) within 22 s. Thus, we adopted this detector for our investigations. Figure 3a shows this study’s CZT detector module (H3D, M400), which was 5.7 × 5.7 × 10.2 cm^3^ and weighed 0.6 kg. Figure 3a also shows that the CZT crystal had dimensions of 4.4 × 4.4 × 1 cm^3^, it was pixelated with 22 × 22 anode and cathode pixels, and it had a depth of interaction (i.e., DOI) that was based on the time it took for the electrons and holes to reach the anodes and cathodes. Therefore, this detector is PSD-capable of providing the x, y, and z coordinates of the interaction location within the detector, as well as the energy deposited at that location. In addition, if gamma rays quickly interacted at two different locations within the detector through Compton scattering, their positions and energies were recorded separately. Figure 3b shows the experimental setup, with the detector and radiation source placed 1 m apart on the ground. For the experiment, we also designed a support structure to enable rotations around two axes that can simulate radiation incidents from all directions. The rotation axes were located at the center of the CZT crystal. The support structure was made entirely of plastic to minimize the Compton effect of gamma rays by minimizing the amount of metal used.

**Figure 4:**
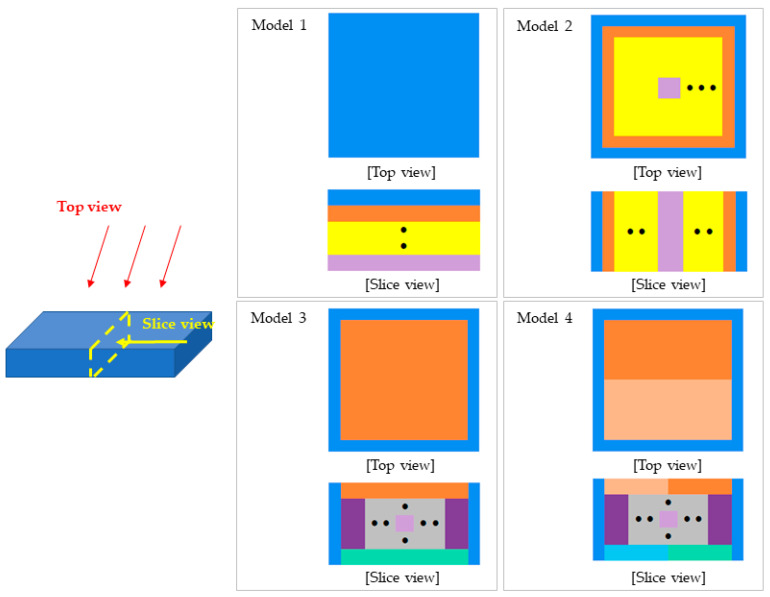
Top view and slice view of 4 models for the evaluation of G(E)_PG_ function.

For evaluating the G(E)_PG_ functions, four different models, divided into 22 × 22 × 10 virtual pixels, were used, as shown in Figure 4. Model 1 had 10 groups in the AP direction, Model 2 had 11 groups in the lateral direction, Model 3 was simultaneously divided into 14 groups in both the AP and lateral directions, and Model 4 unnecessarily divided the AP direction layers into half for verification of dividing rules, resulting in 22 groups.

**Figure 5:**
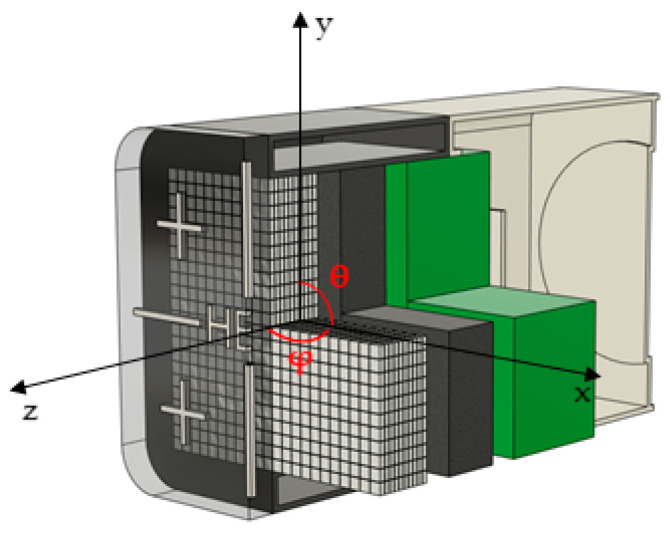
Schematic of the geometry of MCNP6 simulations.

We used a Monte Carlo N−Particle Transport Code (MCNP6.2) to compare and analyze the proposed method (G(E)_PG_ functions) with the conventional G(E) method (G(E)_C_ function). Figure 5 shows a schematic of the MCNP simulation environment. First, the CZT detector was modeled to be the same size as the actual detector (dimensions: 10.2 × 5.7 × 5.7 cm^3^). The detector case was made of aluminum with a thickness of 4 mm and a density of 2.70 g/cm^3^. The CZT crystal comprised Cd(1−x):Zn(x):Te(1) and simulated a density of 5.8 g/cm^3^ (ratio: Cd:Zn:Te = 0.89:0.11:1). Subsequently, to mimic the cooling system and field−programmable gate array circuit, a 10 mm thick aluminum plate (density of 2.70 g/cm^3^) and a 19 mm thick plastic plate (density: 1.13 g/cm^3^) were placed behind the CZT crystal. As shown in Table 1, different datasets were created for optimizing coefficients of G(E)_PG_ functions and coefficients of G(E)_C_ function.

For optimizing A(K) of G(E)_PG_ functions, the pixelated CZT crystal consists of a total of 4840 pixels, arranged in a grid of 22 × 22 × 10 in the x, y, and z directions, with each pixel having a size of 0.2 × 0.2 × 0.1 cm^3^. Each pixel was assigned a pulse height tally (i.e., F8 tally), and a Gaussian−energy broadening filter (GEB) was applied to obtain a spectrum that closely resembles the actual response of the detector. The input parameters of the GEB card were a = 5.704 × 10^−4^, b = 4.753 × 10^−3^, and c = 1.223. To take on the appearance of unknown source distribution, the gamma ray was irradiated from a random location on 4 pi sphere as a circular aligned field that is sufficiently larger than the detector, towards the center of the detector, and the energy of the gamma ray had a random value between 100 keV and 2000 keV. Using the simulation environment described above, 2000 datasets were generated for the optimization of A(K) of G(E)_PG_ functions. The number of A(K) coefficients to be optimized for each model is the product of $K_{MAX}$ and the number of groupings. Therefore, 50 coefficients for model 1, 55 for model 2, 70 for model 3, and 110 for model 4 were optimized.

For optimizing A(K) of G(E)_C_ function, the CZT crystal was composed of a size of 4.4 × 4.4 × 1 cm^3^ with F8 tally and GEB. The gamma ray with an energy between 100 keV and 2000 keV was irradiated from a circular aligned field that was sufficiently larger than the detector in the AP direction. We generated 100 datasets for optimization of A(K) of G(E)_C_ function.

The method of optimization for both G(E)_PG_ functions and G(E)_C_ function was using the ADAM method, which obtained high accuracy of dose estimation in previous studies [5,13]. The ADAM optimizer is an algorithm that can converge to the global minimum in optimization problems with many variables, making it suitable for the optimizing up to 110 variables. We then used the mean absolute percentage error (MAPE) as the loss function to prevent underestimating low−energy gamma rays.

## 3. Results and Discussion

### 3.1. Comparison between the G(E)_PG_ and G(E)_C_ Functions for Dose Estimation

Our investigations compared and verified five models, namely, four proposed G(E)_PG_ models (Figure 4) and one G(E)_C_ model, to evaluate the proposed G(E)_PG_ functions. Then, we constructed an evaluation dataset using 19 energies ranging from 100 keV to 2000 keV on a linear scale for sources located in four directions (i.e., [Φ = 0, θ = 0], [Φ = 0, θ = 180], [Φ = 45, θ = 90], [Φ = 45, θ = 135]).

**Figure 6:**
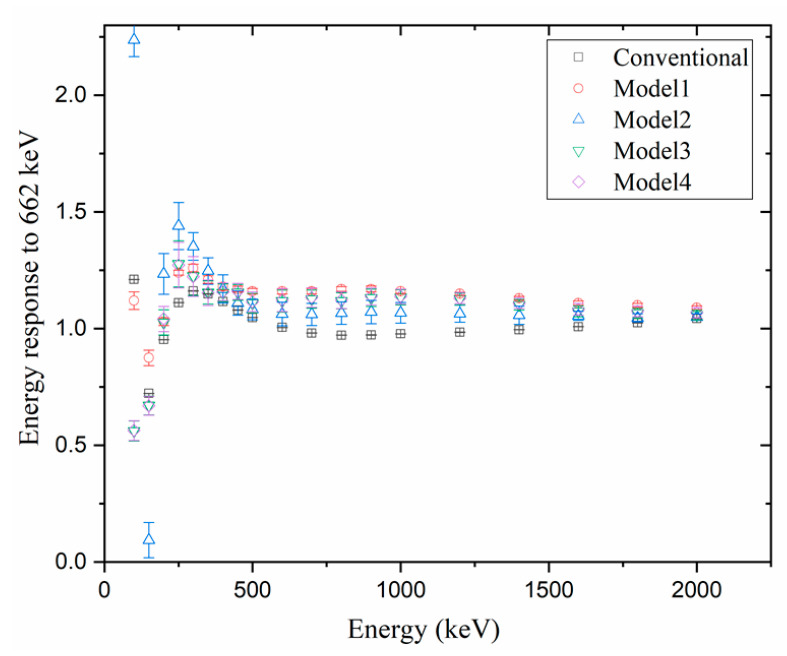
Comparison of the simulation results of normalized energy response to Cs137 in the AP direction using 4 G(E)_PG_ models and 1 G(E)_C_ model under source located at [Φ = 0, θ = 0].

**Figure 7:**
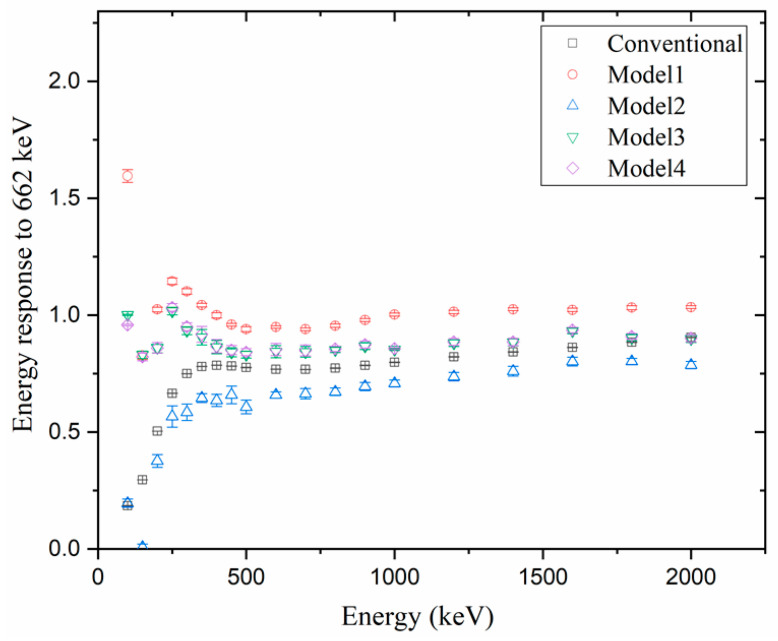
Comparison of the simulation results of normalized energy response to Cs137 in the AP direction using 4 G(E)_PG_ models and 1 G(E)_C_ model under source located at [Φ = 0, θ = 180].

**Figure 8:**
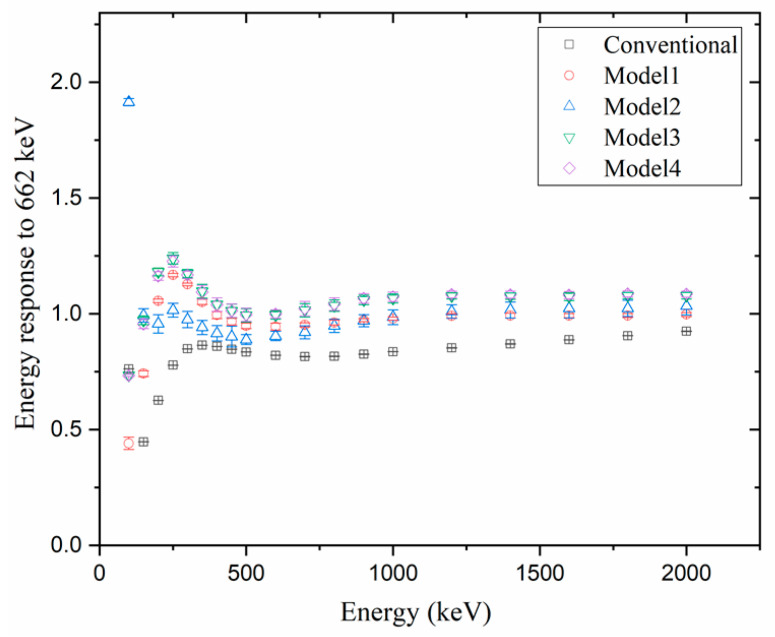
Comparison of the simulation results of normalized energy response to Cs137 in the AP direction using 4 G(E)_PG_ models and 1 G(E)_C_ model under source located at [Φ = 45, θ = 135].

Figure 6, Figure 7 and Figure 8 compare the energy responses normalized to Cs_137_ (i.e., the gamma energy at 662 keV) at [Φ = 0, θ = 0] (i.e., AP). When the energy response was closer to 1, the estimated dose was considered more accurate, and if the energy response was higher than 1, the dose was overestimated; however, if it was lower than 1, the dose was tagged as underestimated.

(3) $$Normalized\;energy\;response\;to\;Cs\;137\;in\;the\;AP\;direction=\frac{\frac{{H^{*}(10)}_{E,Est}}{{H^{*}(10)}_{E,True}}}{\frac{{H^{*}(10)}_{662keV,AP,Est}}{{H^{*}(10)}_{662keV,AP,True}}}$$

where $H^{*}(10)$ denotes the ambient dose equivalent, subscripts 662 keV and E denote the gamma ray energies, and subscripts Est and True denote the estimated values using the G€_PG_ o€(E)_C_ functions and true values, respectively.

As shown in Figure 6, when the source was located at [Φ = 0, θ = 0], the G(E)_C_ function accurately estimated the dose within the entire energy range. This is a reasonable result as the G(E)_C_ model was optimized considering only the case when the source is located at [Φ = 0, θ = 0]. Investigations also revealed that although the G(E)_PG_ Model 2 estimated the dose with similar accuracy to the G(E)_C_ model above 300 keV, its dose estimation accuracy at low energies was substantially lower. For the G(E)_PG_ models 1, 3, and 4, the dose was estimated at energy responses between 0.70 and 1.25, showing a modest error.

Figure 7 and Figure 8 show the energy responses of the five models when the sources were located at [Φ = 0, θ = 180], [Φ = 45, θ = 135], respectively. At the two angles, we observed that the G(E)_C_ function underestimated the doses for all energies. This underestimation was particularly pronounced for energies below 300 keV, because at low energies, most reactions occurred on the CZT crystal’s surface. At two angles, the incident area is reduced compared to the area in the direction of the AP, or the gamma−fluence is shielded by the detector system. While with the G(E)_PG_ Model 2, the error at low energies was too large, or the variability of the error was too high, resulting in decreased reliability, the G(E)_PG_ models 1, 3, and 4 showed slight underestimations in some regions, accurately estimating the dose in most. Among them, when comparing with G(E)_PG_ models 3 and 4, the dose was estimated almost similarly, regardless of the angle and energy. The G(E)_PG_ Model 4 is the one that divided the groups more than Model 3, violating the rules. While the G(E)_PG_ Model 4 was divided into 22 groups, the G(E)_PG_ Model 3 was divided into 14 groups. Consequently, although the G(E)_PG_ Model 4 had 1.57 times more groups, the estimation’s accuracy did not improve. In other words, our investigations confirmed that dividing into more groups did not necessarily improve the estimation’s accuracy, and thus it is meaningful to divide following the rules.

Subsequently, to compare the accuracy of dose estimation for an unknown source distribution where the source location is completely unspecified, 1000 datasets were generated for the five models with random directions of 4 pi and random energies between 50–2000 keV for each dataset. Each dataset was simulated with sufficient (5 × 10^7^) nps. For Cs137 source, 1.5 × 10^6^ particles were recorded for each dataset, with more than 2 × 10^3^ particles per channel, and a total of 1 × 10^5^ particles per group. Consequently, the variance of each channel was low, below 4%. In general, a variance of 5% or less is considered to be statistically small enough in MCNP. Table 2 shows the means and standard deviations of the dose estimation results’ MAPE for the 1000 generated datasets.

An error’s average and standard deviation are important indicators in estimating the dose. If the error’s standard deviation is high, it can be judged that unreliable results were obtained for specific energies or directions. From our study, although the MAPE’s averages for the G(E)_PG_ models 1 and 2 were lower than that of the G(E)_C_ model, their standard deviations were higher. As the G(E)_PG_ Model 4 unnecessarily increased its number of coefficients, resulting in increased computational complexity without improving the dose estimation’s accuracy, it was more appropriate to use the G(E)_PG_ Model 3. Accordingly, with the G(E)_PG_ Model 3, both its MAPE−based average and standard deviation were confirmed to be twice as improved compared with the G(E)_C_ model, meaning that in situations where the direction and distribution of the radiation source had not been specified, Model 3 could provide a more reliable dose estimation than the conventional method.

Table 3, Table 4 and Table 5 compare the simulation and experimental data between the G(E)_PG_ Model 3 and the G(E)_C_ model at the six investigated angles (i.e., [Φ = 0, θ = 0], [Φ = 0, θ = 90], [Φ = 0, θ = 180], [Φ = 45, θ = 45], [Φ = 45, θ = 90], [Φ = 45, θ = 135]) for Cs137, Co60, and Eu152. H*(10)_True_ denotes the true value of the ambient dose equivalent_,_ H*(10)_C_ and H*(10)_PG_ denote the estimated values using G(E)_C_ model and G(E)_PG_ Model 3, and subscripts Simul and Ex denote the estimated values using the simulation and experimental data. The experimental results confirmed that the G(E)_C_ model estimated the dose accurately in the AP direction (i.e., [Φ = 0, θ = 0]). However, except for the AP direction, G(E)_PG_ Model 3 estimated the dose more accurately than the G(E)_C_ model, especially in the three cases of Φ = 45. The G(E)_C_ model overestimated the dose in the case of a wide incident surface (e.g., [Φ = 45, θ = 45]), and underestimated the dose in the case of a narrow incident surface (e.g., [Φ = 0, θ = 90]). G(E)_PG_ Model 3 estimated the dose regardless of the area of incident surface because multiple functions complement each other to estimate the dose. Consequently, while the average MAPE for the experimental results using the G(E)_C_ model was estimated to be 20.1 ± 1.4, 22.4 ± 2.3, and 13.2 ± 2.0 for Cs_137_, Eu_152_, and Co_60_, respectively, the average MAPE for the experimental results using G(E)_PG_ Model 3 was estimated to be 12.7 ± 3.5, 9.5 ± 3.9, and 8.8 ± 3.6, respectively, achieving an improvement of more than 1.5 times.

### 3.2. Evaluation of the G(E)_PG_ Functions’ Dose Contributions

**Figure 9:**
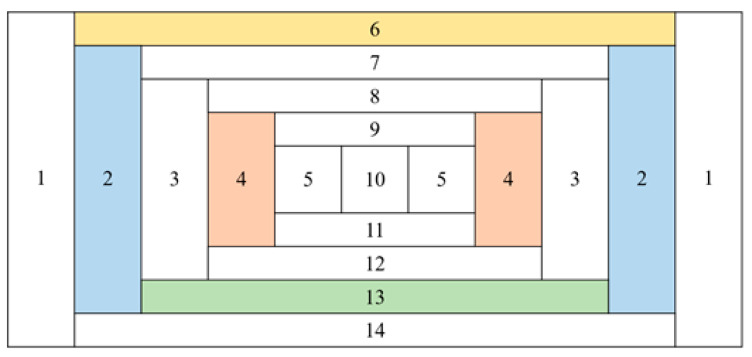
Layout of the group number for G(E)_PG_ Model 3: Group 2 (blue), Group 4 (red), Group 6 (yellow), and Group 13 (green).

**Figure 10:**
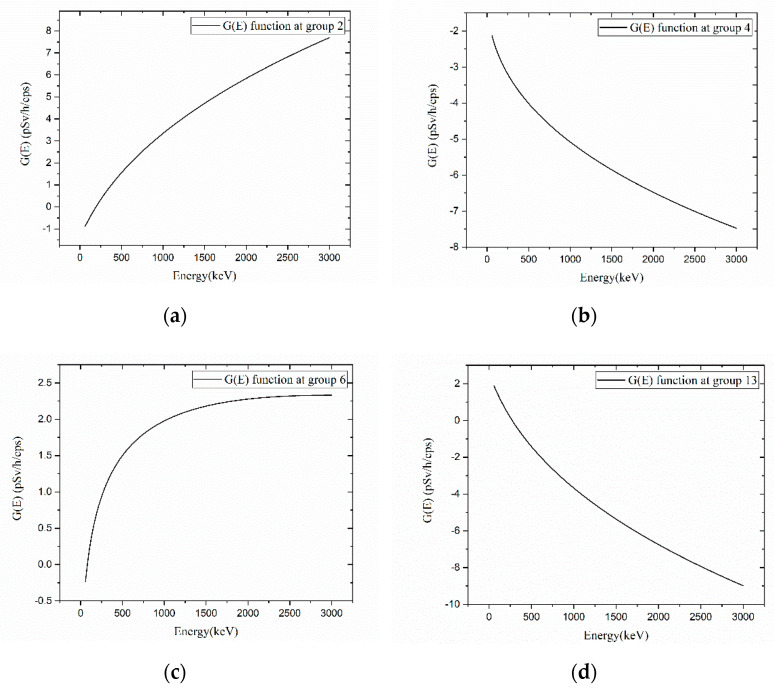
G(E) function of G(E)_PG_ Model 3: (**a**) G(E) function at Group 2; (**b**) G(E) function at Group 4; (**c**) G(E) function at Group 6; (**d**) G(E) function at Group 13.

When comparing the G(E)_PG_ function with the G(E)_C_ functions, intuitively understanding their dose estimation methods is challenging because multiple G(E)_PG_ functions corresponding to each group complement each other. Accordingly, while Figure 9 shows a slice view of the G(E)_PG_ Model 3 with the numbers assigned to each group, Figure 10 shows the G(E)_PG_ functions assigned to Groups 2, 4, 6, and 13. In Group 2, the dose was reduced when the accumulated energy of the detector was approximately below 200 keV, and increased above 200 keV. Conversely, unlike Group 2, Group 13′s G(E) function increased the dose for energy ranges of approximately 200 keV or less and decreased the dose for energy ranges above 200 keV. In Group 4, it reduced the doses in all energy ranges but played opposite roles in Group 6. Thus, it was possible to confirm that not a specific group estimated the dose at a specific energy but multiple groups were involved in the dose estimation.

**Figure 11:**
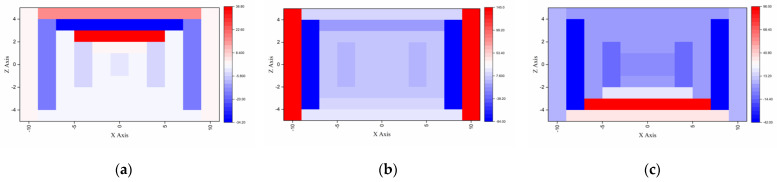
G(E)_PG_ functions’ dose contributions for estimating doses at 662 keV: (**a**) source located at [Φ = 0, θ = 0]; (**b**) source located at [Φ = 0, θ = 90]; (**c**) source located at [Φ = 0, θ = 180].

Figure 11 represents how much each group contributed to estimating the gamma ray doses at 122 keV (Eu_152_) and three different locations ([Φ = 0; θ = 0], [Φ = 0; θ = 90], [Φ = 0; θ = 180]). For the low−energy gamma rays, although all groups contributed to estimating the dose, the 2–3 groups on the surface in the incidence direction particularly contributed significantly to determining the dose. This result is reasonable, considering that most of the reactions occurred on the surface when the gamma ray’s energy was low.

**Figure 12:**
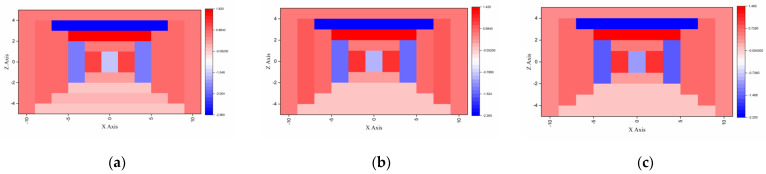
G(E)_PG_ functions’ dose contributions for estimating doses at 122 keV: (**a**) source located at [Φ = 0, θ = 0]; (**b**) source located at [Φ = 0, θ = 90]; (**c**) source located at [Φ = 0, θ = 180].

Figure 12 shows the contribution of each group when estimating doses at 662 keV (Cs_137_). Unlike low−energy gamma rays, although all groups contributed relatively evenly to dose estimations, and no group contributed distinctly, a slight difference in contribution depending on the direction was observed. Even this slight difference in contribution enables accurate dose estimation compared to conventional G(E_)C_ function, regardless of the direction of the source.

Previous studies have focused on estimating the direction of the radiation source using the reaction position and energy with the use of PSD. In particular, studies estimating the position of gamma ray source using collimator structures or Compton scattering have been predominant. To derive consistent operational quantities independent of the direction of radiation sources, existing methods require two steps: locating the position and adjusting the measured values based on the position. However, these two steps can lead to cumulative errors and require significant computational time or additional physical structures. In this study, the proposed method aims to estimate operational quantities without determining the location, through grouping and a compensation function called G(E) function. The proposed method is a novel approach that estimates an ambient dose equivalent parameter {H*(10)} with high accuracy and a small computational load by using the differences in response to each group inside the detector, without determining the location of the radiation source.

## 4. Conclusions

For dose estimation, this manuscript proposes a new method that adopts pixel−grouping multiple G(E) functions through a PSD detector. First, to reduce unnecessary divisions when grouping the pixels, we established three rules and decided on four models to validate them. Next, we used MCNP to simulate the incident gamma ray in the 4 pi direction, divide the CZT crystals into 22 × 22 × 10, and group them to optimize the G(E)_PG_ function’s models and verify the dose estimation accuracies for the G(E)_PG_ functions. For comparisons with G(E)_PG_ functions, we simulated the conventional method having only one G(E)_C_ function separately. Investigations confirmed that, except for the source located at [Φ = 0, θ = 0] (i.e., AP direction), the G(E)_PG_ functions could estimate doses more accurately than the G(E)_C_ function. For the unknown source distribution scenario and at random directions/energies between 50 and 2000 keV, the proposed method showed an improvement of approximately two times compared with the conventional method regarding the average and standard deviation of MAPE for the 1000 simulation data. Furthermore, for the experiments with three sources (i.e., Cs_137_, Eu_152_, Co_60_) and at six locations (i.e., [Φ = 0, θ = 0], [Φ = 0, θ = 90], [Φ = 0, θ = 180], [Φ = 45, θ = 45], [Φ = 45, θ = 90], [Φ = 45, θ = 135]), it was also verified that the proposed method provided more than 1.5 times improvement compared with the conventional method. Therefore, this study’s proposed method provided accurate, consistent, and reliable dose estimations regardless of the source’s direction and energy. By applying the proposed method, it will be possible to estimate the workers’ dose more accurately and protect them from radiation exposure in situations where the location of the radiation source is unknown (e.g., investigation of radioactive contamination areas and decommissioning of nuclear power plants). We believe that this study will not only estimate an ambient dose equivalent parameter {H*(10)} but will also contribute to estimating operational quantities (e.g., fluence, flux, and air kerma).

## Figures and Tables

**Table 1:** Simulation conditions for the optimization of the G(E)_PG_ functions and the G(E)_C_ function.

Parameters	The G(E)_PG_ Functions	The G(E)_C_ Function
# of tallies	4840 (22 × 22 × 10)	1
Source direction	Random direction	AP direction
Energies of the gamma rays	100–2000 keV	100–2000 keV
# of dataset	2000	100
# of A(K) coefficients	5 × 10 (Model 1) 5 × 11 (Model 2) 5 × 14 (Model 3) 5 × 22 (Model 4)	5

**Table 2:** Evaluation results of G(E)_PG_ models and G(E)_C_ model for 1000 datasets randomly distributed at 4 pi.

	Average MAPEs (%)	Standard Deviation of MAPE
Conventional Model	11.81	10.40
Model 1	7.05	11.56
Model 2	9.89	14.41
Model 3	6.36	5.85
Model 4	6.30	5.79

**Table 3:** Simulation and experimental results of G(E)_PG_ Model 3 and G(E)_C_ model at six angles ([Φ = 0, θ = 0], [Φ = 0, θ = 90], [Φ = 0, θ = 180], [Φ = 45, θ = 45], [Φ = 45, θ = 90], [Φ = 45, θ = 135]) for Cs_137_.

Cs_137_	H*(10)_True_	H*(10)_C,Simul_	MAPE	H*(10)_PG,Simul_	MAPE	H*(10)_C,Ex_	MAPE	H*(10)_PG,Ex_	MAPE
[Φ = 0, θ = 0]	43.7	43.1 ± 0.7	1.4 ± 1.6	47.6 ± 1.1	8.9 ± 2.5	39.1 ± 0.9	10.5 ± 2.1	48.5 ± 2.1	11.0 ± 4.8
[Φ = 0, θ = 90]		33.8 ± 0.9	22.7 ± 2.1	36.2 ± 1.6	17.2 ± 3.7	30.2 ± 0.5	30.9 ± 1.1	33.3 ± 1.4	23.8 ± 3.2
[Φ = 0, θ = 180]		33.2 ± 0.6	24.0 ± 1.4	35.6 ± 1.4	18.5 ± 3.2	30.7 ± 0.4	29.7 ± 0.9	36.1 ± 1.2	17.4 ± 2.7
[Φ = 45, θ = 45]		47.9 ± 0.4	9.6 ± 0.9	45.4 ± 1.8	3.9 ± 4.1	42.2 ± 0.5	3.4 ± 1.1	40.2 ± 1.9	8.0 ± 4.3
[Φ = 45, θ = 90]		35.7 ± 0.6	18.3 ± 1.4	43.2 ± 1.2	1.1 ± 2.7	30.4 ± 0.7	30.4 ± 1.6	38.1 ± 1.6	12.8 ± 3.7
[Φ = 45, θ = 135]		36.0 ± 1.0	17.6 ± 2.3	43.8 ± 1.3	0.2 ± 3.0	36.9 ± 0.7	15.6 ± 1.6	42.2 ± 1.1	3.4 ± 2.5
Average	43.7	38.3 ± 0.7	15.6 ± 1.6	42.0 ± 1.4	8.3 ± 3.2	34.9 ± 0.6	20.1 ± 1.4	39.7 ± 1.6	12.7 ± 3.5

**Table 4:** Simulation and experimental results of G(E)_PG_ Model 3 and G(E)_C_ model at six angles ([Φ = 0, θ = 0], [Φ = 0, θ = 90], [Φ = 0, θ = 180], [Φ = 45, θ = 45], [Φ = 45, θ = 90], [Φ = 45, θ = 135]) for Eu_152_.

Eu_152_	H*(10)_True_	H*(10)_C,Simul_	MAPE	H*(10)_PG,Simul_	MAPE	H*(10)_C,Ex_	MAPE	H*(10)_PG,Ex_	MAPE
[Φ = 0, θ = 0]	80.5	80.6 ± 1.9	0.1 ± 2.4	89.1 ± 3.1	10.7 ± 3.9	86.9 ± 3.9	8.0 ± 4.8	91.8 ± 4.5	14.0 ± 5.6
[Φ = 0, θ = 90]		64.6 ± 3.1	19.8 ± 3.9	68.4 ± 3.0	15.0 ± 3.7	55.4 ± 0.6	31.2 ± 0.7	72.1 ± 2.6	10.4 ± 3.2
[Φ = 0, θ = 180]		63.5 ± 1.5	21.1 ± 1.9	72.7 ± 2.1	9.7 ± 2.6	54.5 ± 1.1	32.3 ± 1.4	85.7 ± 2.4	6.5 ± 2.9
[Φ = 45, θ = 45]		89.5 ± 1.0	11.2 ± 1.2	81.6 ± 2.7	1.4 ± 3.4	90.4 ± 1.8	12.3 ± 2.2	72.7 ± 2.2	9.7 ± 2.8
[Φ = 45, θ = 90]		67.9 ± 1.7	15.7 ± 2.1	78.0 ± 3.5	3.1 ± 4.3	54.1 ± 1.6	32.8 ± 2.0	76.5 ± 4.1	5.0 ± 5.0
[Φ = 45, θ = 135]		66.8 ± 2.4	17.0 ± 3.0	84.8 ± 3.3	5.3 ± 4.1	66.1 ± 2.0	17.9 ± 2.5	89.7 ± 3.0	11.4 ± 3.7
Average	80.5	72.2 ± 1.9	14.1 ± 2.4	79.1 ± 3.0	7.5 ± 3.7	67.9 ± 1.8	22.4 ± 2.3	81.4 ± 3.1	9.5 ± 3.9

**Table 5:** Simulation and experimental results of G(E)PG Model 3 and G(E)C model at six angles ([Φ = 0, θ = 0], [Φ = 0, θ = 90], [Φ = 0, θ = 180], [Φ = 45, θ = 45], [Φ = 45, θ = 90], [Φ = 45, θ = 135]) for Co60.

Co_60_	H*(10)_True_	H*(10)_C,Simul_	MAPE	H*(10)_PG,Simul_	MAPE	H*(10)_C,Ex_	MAPE	H*(10)_PG,Ex_	MAPE
[Φ = 0, θ = 0]	139.2	137.8 ± 2.7	1.0 ± 1.9	149.9 ± 6.4	7.7 ± 4.6	131.7 ± 2.3	5.4 ± 1.6	155.5 ± 7.9	11.7 ± 5.7
[Φ = 0, θ = 90]		118.5 ± 1.3	14.9 ± 0.9	129.3 ± 3.8	7.1 ± 2.7	113.7 ± 1.6	18.3 ± 1.1	123.1 ± 3.7	11.6 ± 2.6
[Φ = 0, θ = 180]		113.9 ± 2.9	18.2 ± 2.1	123.0 ± 4.8	11.6 ± 3.4	112.6 ± 3.7	19.1 ± 2.7	141.6 ± 4.0	1.7 ± 2.9
[Φ = 45, θ = 45]		151.7 ± 2.1	9.0 ± 1.5	140.2 ± 3.2	0.7 ± 2.3	144.4 ± 1.9	3.7 ± 1.4	157.3 ± 4.0	13.0 ± 2.9
[Φ = 45, θ = 90]		122.0 ± 3.8	12.4 ± 2.7	145.9 ± 5.1	4.8 ± 3.7	114.5 ± 4.0	17.7 ± 2.9	122.6 ± 5.1	11.9 ± 3.7
[Φ = 45, θ = 135]		119.1 ± 3.8	14.4 ± 2.7	148.8 ± 4.7	6.9 ± 3.4	118.1 ± 3.5	15.2 ± 2.5	135.2 ± 4.9	2.9 ± 3.5
Average	139.2	127.2 ± 2.8	11.6 ± 2.0	139.5 ± 4.7	6.5 ± 3.4	122.5 ± 2.8	13.2 ± 2.0	139.2 ± 4.9	8.8 ± 3.6
